# Author Correction: Spatial transcriptomics reveals regionally altered gene expression that drives retinal degeneration

**DOI:** 10.1038/s42003-025-08451-8

**Published:** 2025-07-07

**Authors:** Ulrike Schumann, Lixinyu Liu, Riemke Aggio-Bruce, Adrian V. Cioanca, Artur Shariev, Michele C. Madigan, Krisztina Valter, Jiayu Wen, Riccardo Natoli

**Affiliations:** 1 The John Curtin School of Medical Research, The Australian National University, Canberra, ACT Australia; 2 The Shine-Dalgarno Centre for RNA Innovation, The John Curtin School of Medical Research, The Australian National University, Canberra, ACT Australia; 3 The Save Sight Institute, The University of Sydney, Sydney, NSW Australia; 4 The Centre for Computational Biomedical Sciences, The John Curtin School of Medical Research, The Australian National University, Canberra, ACT Australia; 5 ARC Centre of Excellence for the Mathematical Analysis of Cellular Systems (MACSYS), The Australian National University, Canberra, ACT Australia; 6 The School of Medicine and Psychology, The Australian National University, Canberra, ACT Australia; 7 The School of Optometry and Vision Science, The University of New South Wales, Sydney, NSW Australia

**Keywords:** Macular degeneration, Transcriptomics

Correction to: *Communications Biology* 10.1038/s42003-025-07887-2, published online 18 April 2025

In the original version of this Article, the corresponding author Riccardo Natoli was inadvertently omitted. The correct corresponding authors for this article are Riccardo Natoli (riccardo.natoli@anu.edu.au), Jiayu Wen (jiayu.wen@anu.edu.au), and Ulrike Schumann (ulrike.schumann@anu.edu.au). This has now been corrected in both the PDF and HTML versions of the Article.

