# Supplementary material for: Spatial transcriptomics reveals regionally altered gene expression that drives retinal degeneration
Source: Commun Biol. 2025 Apr 18;8:629. doi: 10.1038/s42003-025-07887-2 (PMC12008306; doi:10.1038/s42003-025-07887-2)
Supplement: Supplementary file 1 — Supplementary Information [file 42003_2025_7887_MOESM1_ESM.pdf]

Spatial transcriptomics reveals regionally altered gene expression that drives retinal degeneration.

Ulrike Schumann[1,2,3*], Lixinyu Liu[1,2,4*], Riemke Aggio-Bruce[1,5], Adrian V. Cioanca[1,5], Artur Shariev[1], Michele C. Madigan[3,6], Krisztina Valter[1,5], Jiayu Wen[1,2,4,#], Riccardo Natoli[1,2,5,#]

[1] The John Curtin School of Medical Research, The Australian National University, Canberra, Australia

[2] The Shine Dalgarno Centre for RNA Innovation, The John Curtin School of Medical Research, The Australian National University, Canberra, Australia

[3] The Save Sight Institute, The University of Sydney, Sydney, Australia

[4] The Centre for Computational Biomedical Sciences, The John Curtin School of Medical Research, The Australian National University, Canberra, Australia

[5] The School of Medicine and Psychology, The Australian National University, Canberra, Australia

[6] The School of Optometry and Vision Science, Medicine and Health, The University of New South Wales, Sydney, Australia

[*] equal contribution

[#] corresponding authors

Supplementary Figure S1:

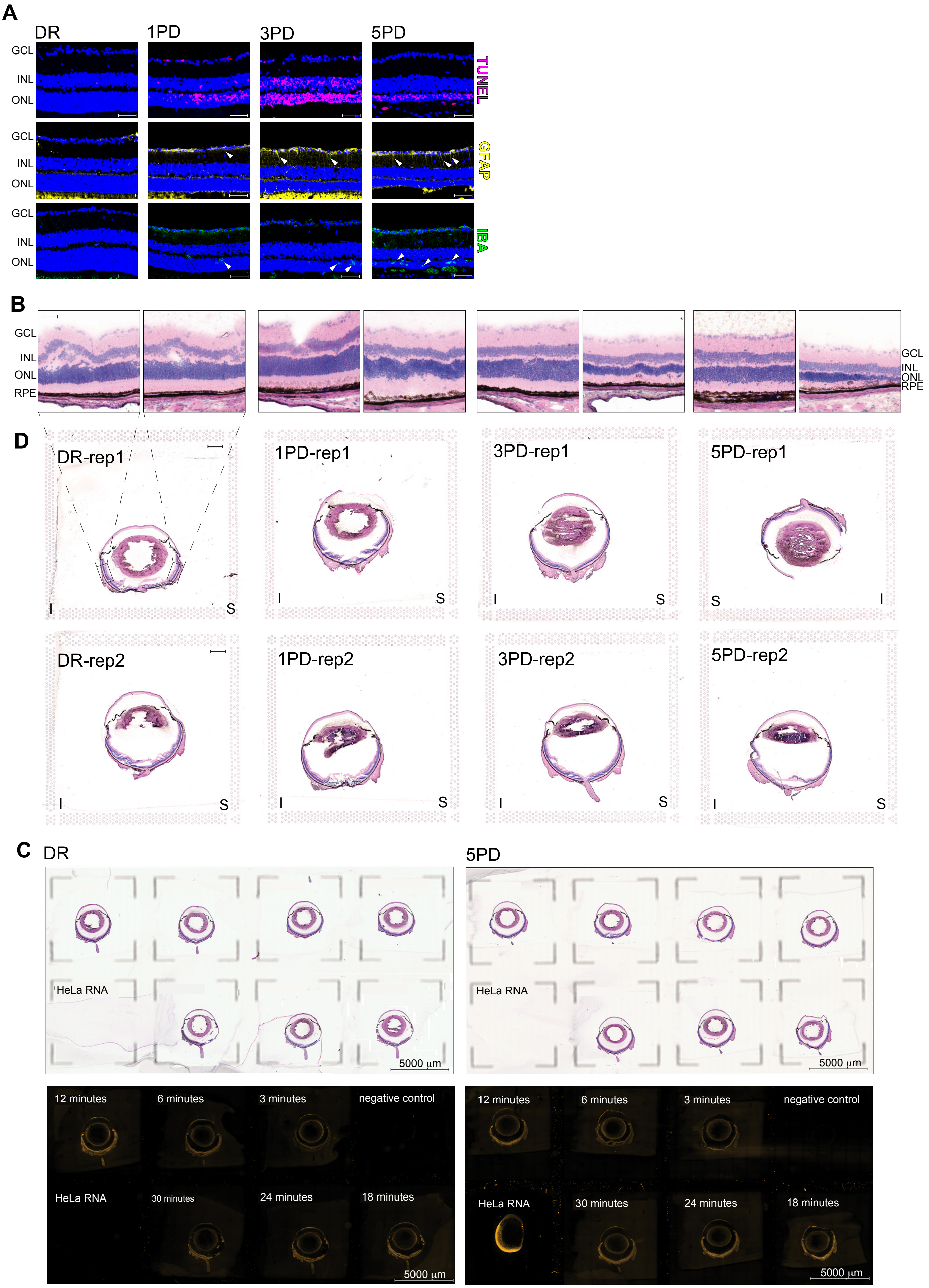

Supplementary Figure S1: (A) Following the PD paradigm, eyes were enucleated, fixed in 4% PFA and embedded in optimal temperature cutting (OCT) medium. 10µm parasagittal sections (including the optic nerve head) were analysed for cell death (TUNEL labelling; top panel), glial cell activation [GFAP (Invitrogen, MA5-12023); middle panel] and immune cell infiltration [IBA (Aves Labs, IBA1-0020); bottom panel] as previously described [1,2]. Sections were counterstained with Hoechst (blue) and imaged using a Nikon confocal microscope at 20x magnification. Representative images of two biological replicate sections are shown. Scale bar = 50µm **(B)** Representative zoomed-in images of the inferior (left) and superior (right) retina for each time point, illustrating thinning of the ONL in the superior retina across the time course. One replicate per time point is shown and the zoomed-in regions indicated by boxes. GCL – ganglion cell layer, INL – inner nuclear layer, ONL – outer nuclear layer, RPE – retinal pigmented epithelium. Scale bar = 50µm **(C)** Top panels: H&E-stained images of the DR control (left) and 5PD (right) tissue sections on the Visium Tissue Optimisation slides (10X Genomics). HeLa total RNA (1 µg) was used as positive control. Bottom panels: Cy3 fluorescent cDNA signal generated from the RNA captured in each tissue section for DR control (left) and 5PD (right) sections. Tissue Optimisation according to manufacturer's instructions using the indicated permeabilisation times. The negative control tissue was not permeabilised. **(D)** H&E-stained images of the Visium Spatial Transcriptomics slides (10X Genomics). A single parasagittal section (including the optic nerve head) from each time point was placed in each capture area and slides processed according to manufacturer's instructions. Two biological replicates from two independent mice were prepared for each time point. Tissue placement for the second replicate was faulty and the slides were subjected to tissue removal according to manufacturer protocols before repeating tissue placement and completing the workflow. Scale bar = 500µm. All images were obtained using a Zeiss AxioScan S1 with

imaging settings recommended by the manufacturer. All capture areas within a single slide were imaged at the same settings. DR – dim reared control, PD – photo-oxidative damage.

Supplementary Figure S2:

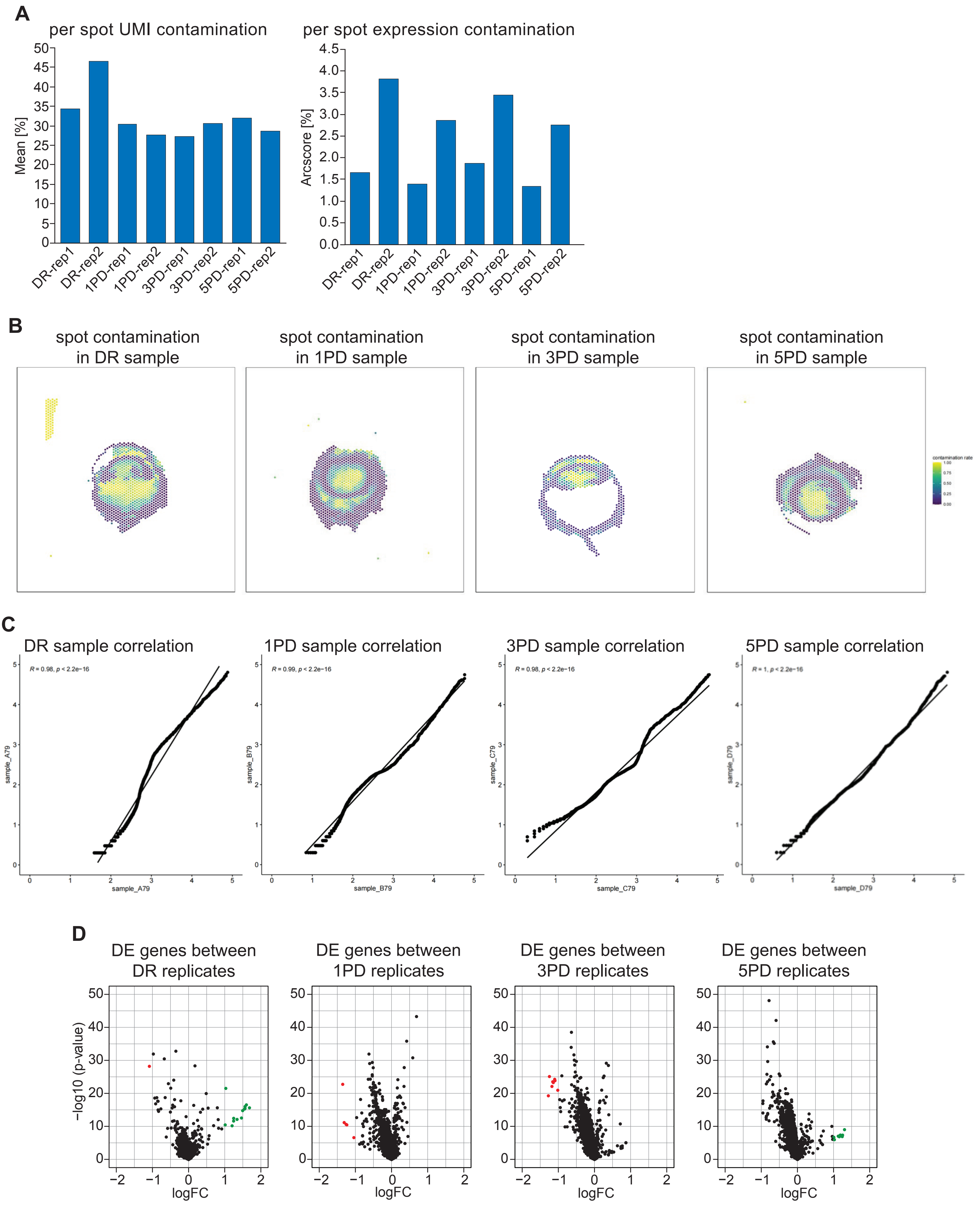

Supplementary Figure S2: (A) SpotClean [3] analysis showing per spot UMI contamination (left) and the per spot contribution of contaminating reads to the gene expression profile (right) in each sample. **(B)** Spots with high contamination levels (heatmap) overlapped the vitreous, anterior chamber, the lens, or are located outside of the tissue. **(C)** Gene expression correlation analysis between the two replicate sample for each time point. **(D)** Volcano plot showing differential gene expression between the two replicate samples for each time point with significantly differentially expressed genes ($p \leq 0.05$, FC ≥ 2) shown in red (downregulated) and green (upregulated). DR – dim reared control, PD – photo-oxidative damage.

Supplementary Figure S3:

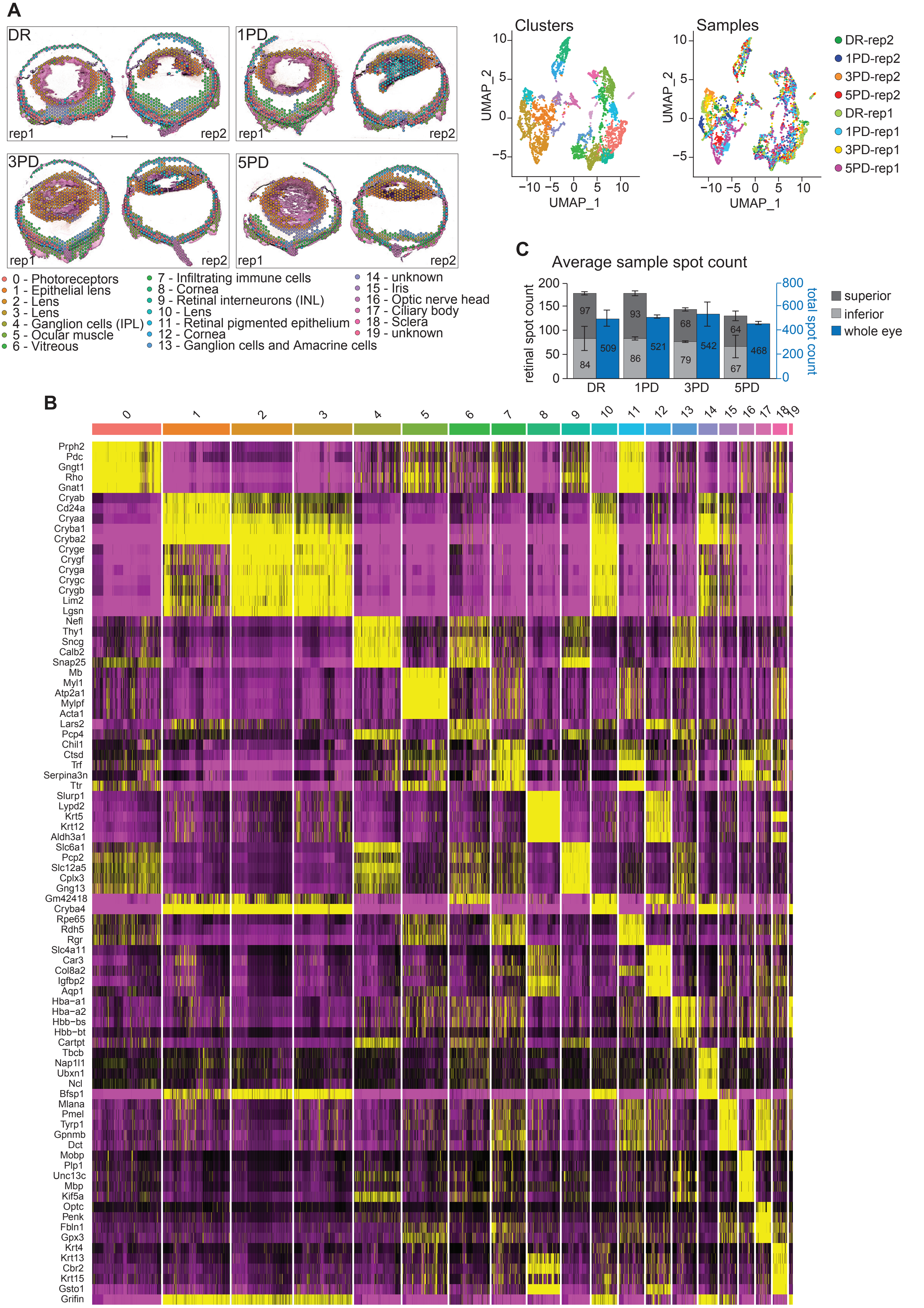

Supplementary Figure S3: (A) Spatial location of the 20 expression clusters identified by unsupervised clustering using STUtility [4] (left). Spots are coloured according to their cluster identity, which is indicated below. Scale bar = 500µm. Reduced dimension presentation (UMAP) of the identified clusters coloured according to clusters identity (middle) or sample (right). **(B)** Heatmap showing the top five differentially expressed genes across all 20 cluster, which was used to assess gene expression characteristics for each cluster and aid cluster identity determination. **(C)** Bar charts showing average tissue coverage with expression spots for each sample indicating the whole tissue in blue and retina/choroid in grey. Error bars indicate standard deviation. Average counts are shown in each bar. DR – dim reared control, PD – photo-oxidative damage.

Supplementary Figure S4:

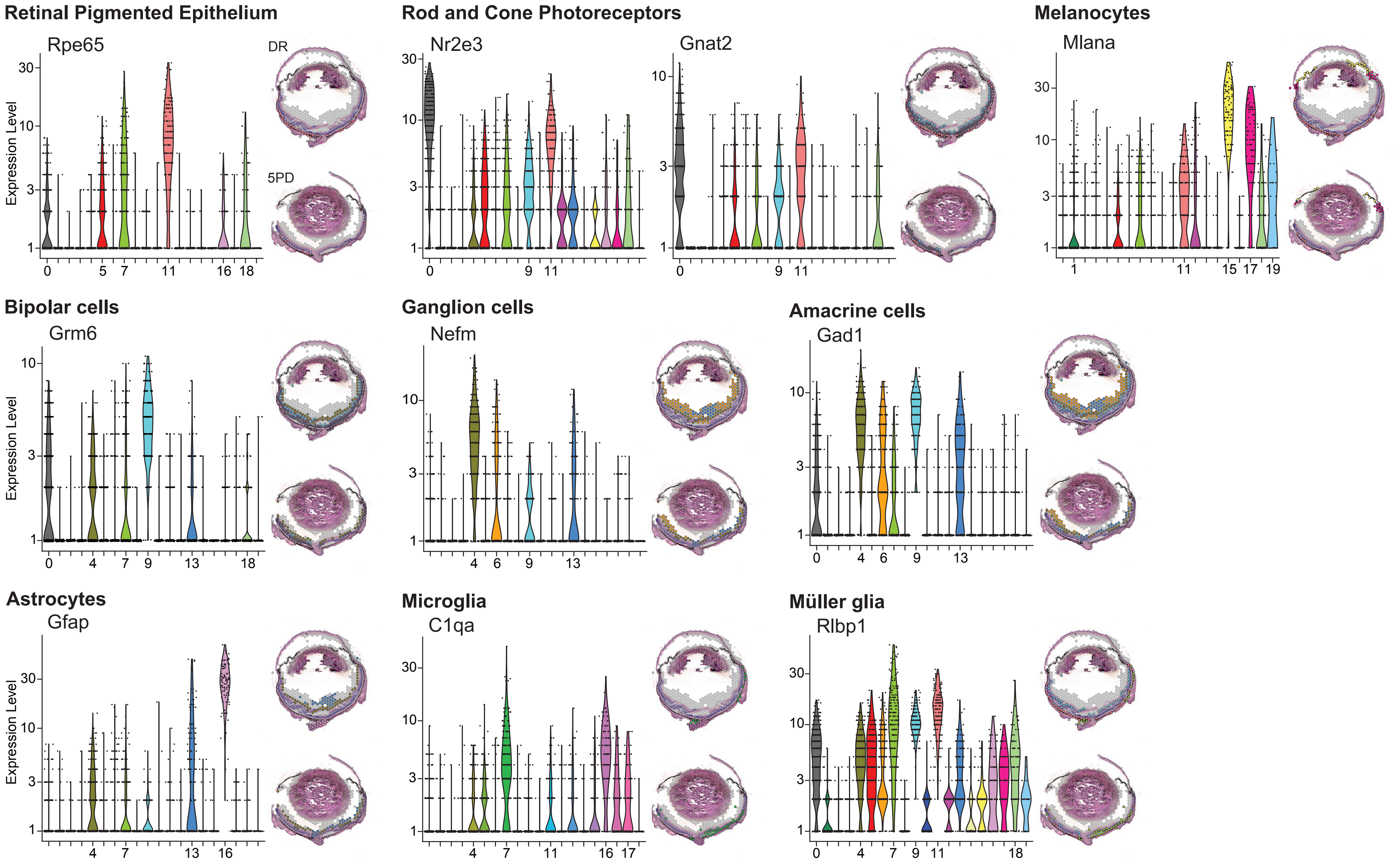

Supplementary Figure S4: Expression level of retinal cell type-specific genes (Supplementary Table S2) within each of the 15 spot clusters identified by STUtility (Supplementary Figure S3). Spatial location of the spots contained within clusters with high expression levels of the indicated genes are shown for one representative DR and 5PD sample. DR – dim reared control, PD – photo-oxidative damage.

Supplementary Figure S5:

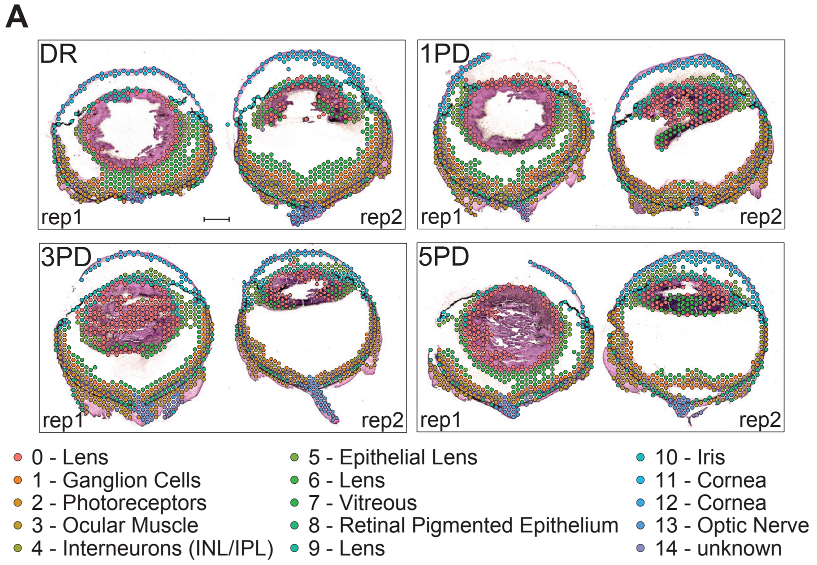

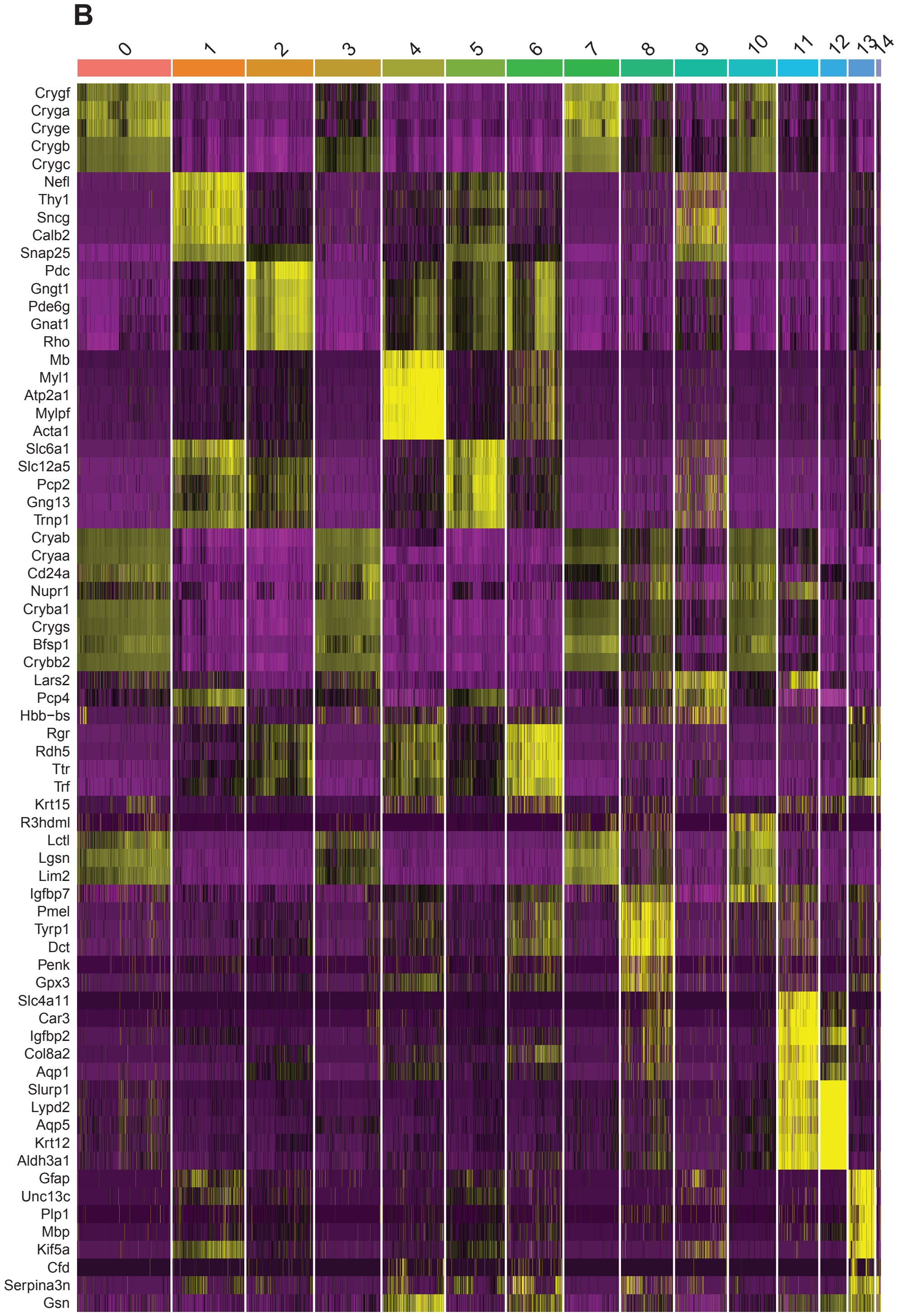

Supplementary Figure S5: (A) Spatial location of the 15 expression clusters identified by unsupervised clustering incorporating morphological information from H&E images using stLearn [5]. Spots are coloured according to their cluster identity, which is indicated below. Scale bar = 500µm. **(B)** Heatmap showing the top five differentially expressed genes across all 15 cluster, which was used to assess gene expression characteristics for each cluster and aid cluster identity determination. DR – dim reared control, PD – photo-oxidative damage.

Supplementary Figure S6:

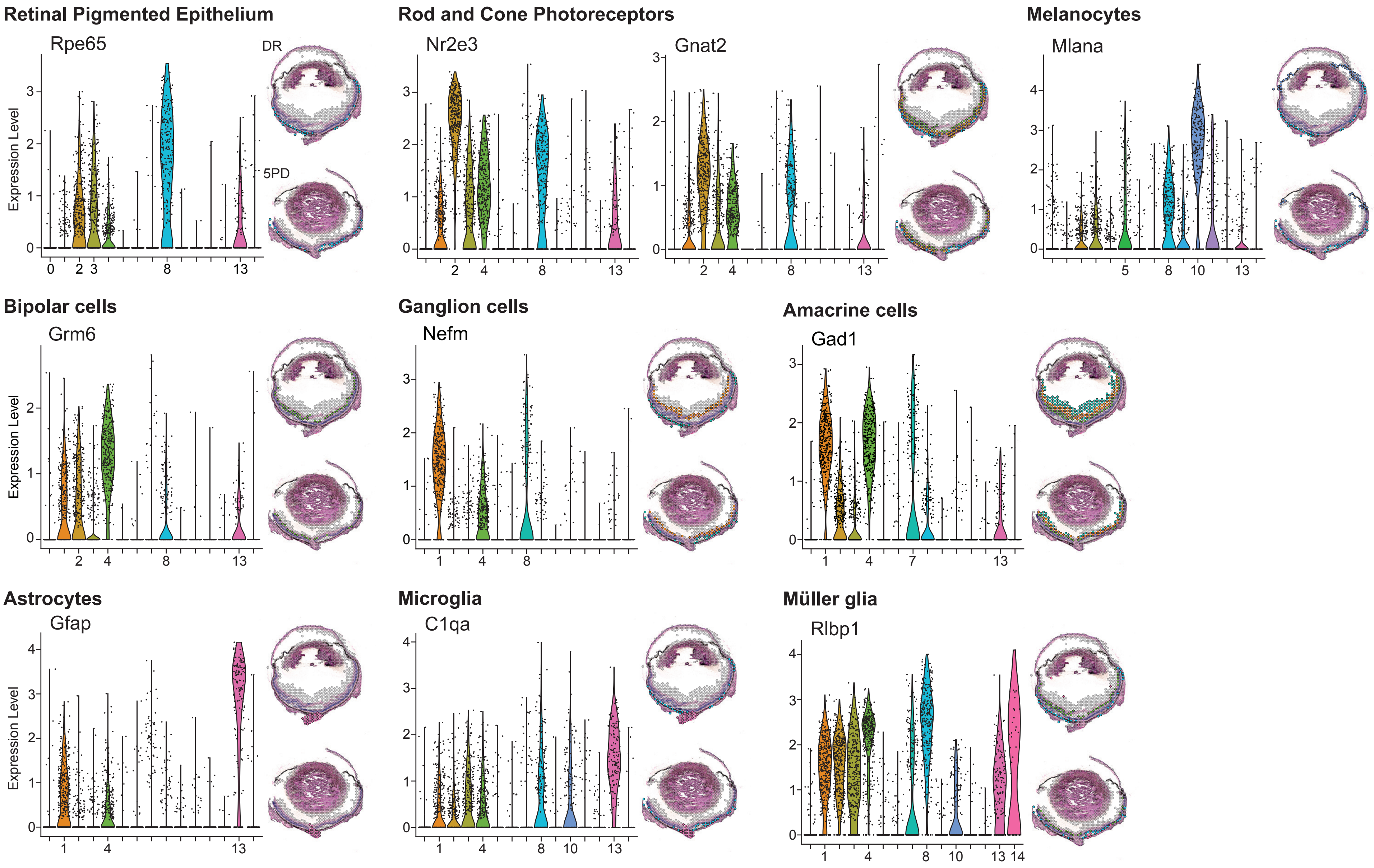

Supplementary Figure S6: Expression level of retinal cell type-specific genes (Supplementary Table S2) within each of the 15 spot clusters identified by stLearn (Supplementary Figure S5). Spatial location of the spots contained within clusters with high expression levels of the indicated genes are shown for one representative DR and 5PD sample. DR – dim reared control, PD – photo-oxidative damage.

Supplementary Figure S7:

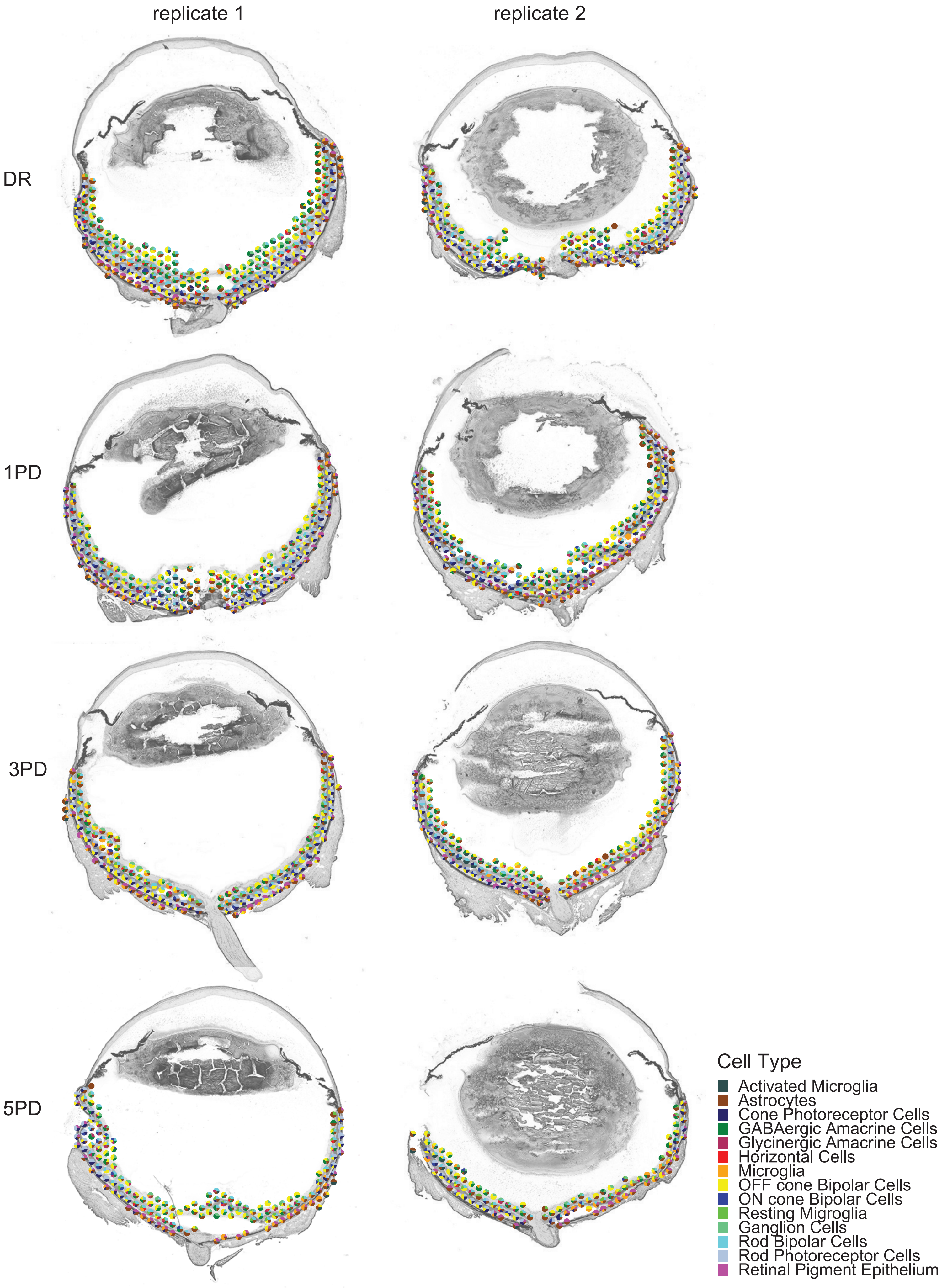

Supplementary Figure S7: Scatter pie plots showing cell type-specific gene expression [6] percentage within each spot of the four retinal/choroid layers for all eight samples. DR – dim reared control, PD – photo-oxidative damage.

Supplementary Figure S8:

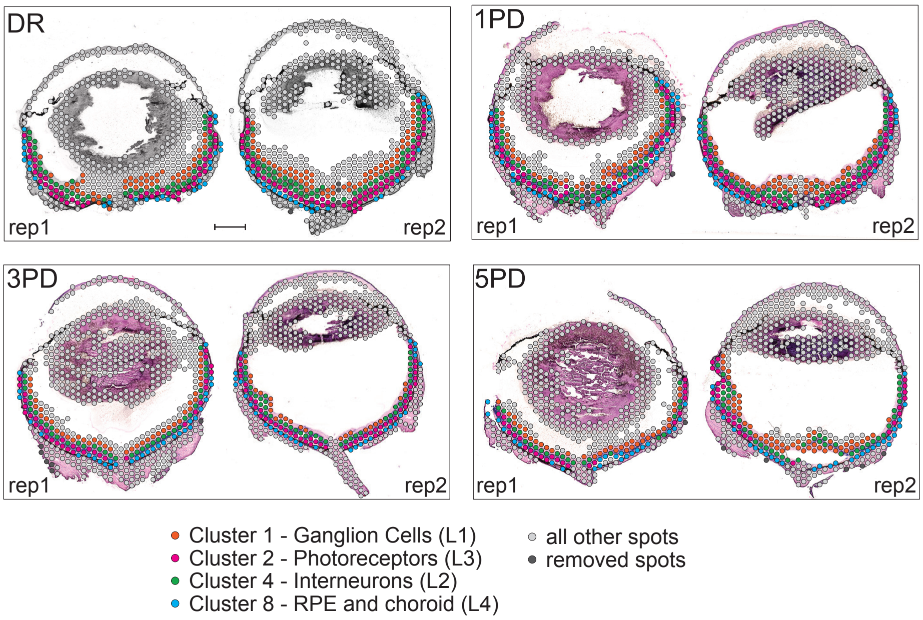

Supplementary Figure S8: Distribution of the four clusters overlapping with the four retinal/choroid layers in all eight samples. Spots clearly outside of the retina/choroid were removed for the analyses and are coloured dark grey. Spots not overlapping the retina/choroid are coloured light grey. Scale bar = 500µm. DR – dim reared control, PD – photo-oxidative damage.

Supplementary Figure S9:

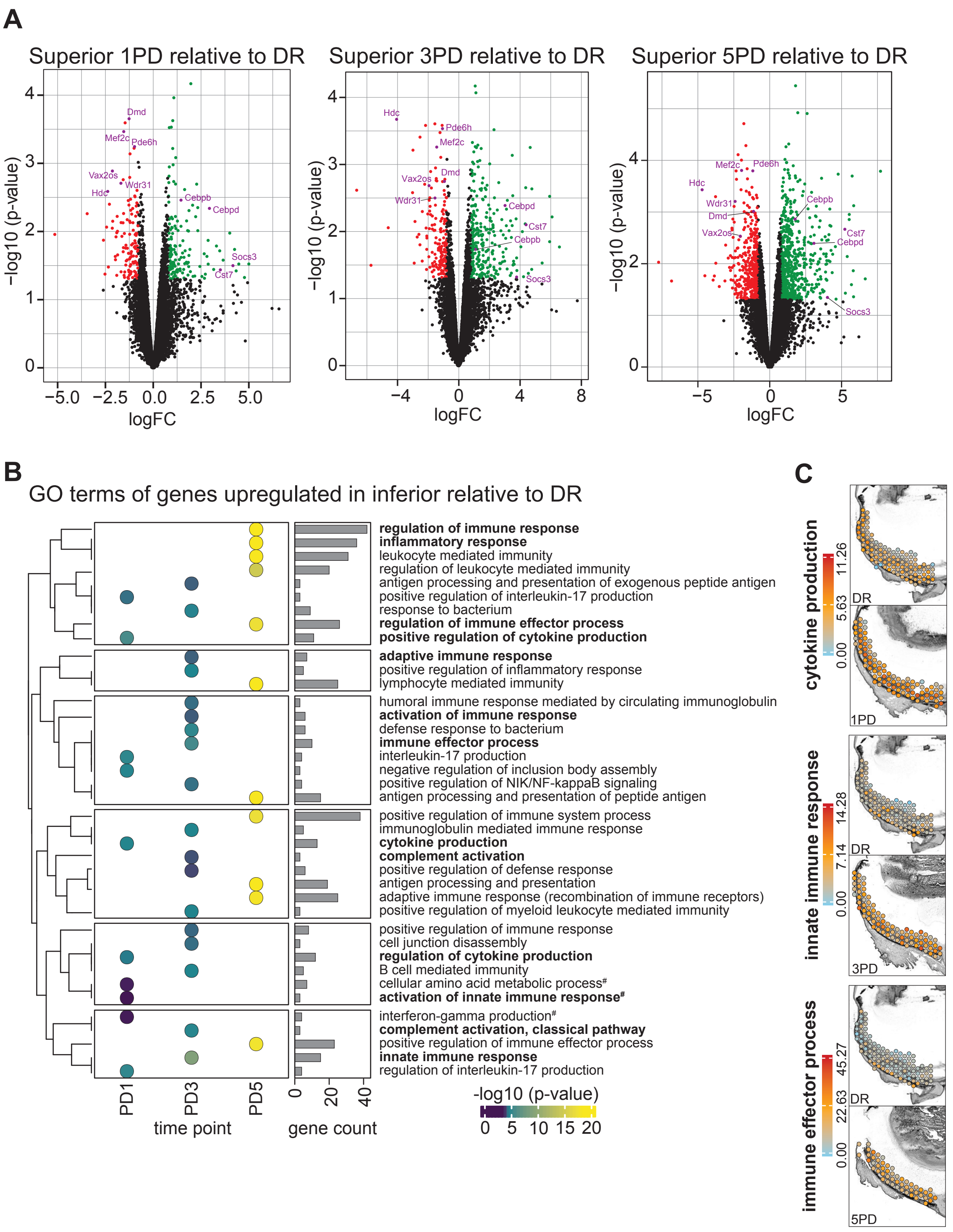

Supplementary Figure S9: (A) Volcano plots showing differential gene expression in the superior retina at each time point compared to DR controls with significantly differentially expressed genes ($p \leq 0.05$, FC ≥ 1.5; Supplementary Table S6A) coloured green (upregulated) and red (downregulated). **(B)** GO term enrichment analysis (Supplementary Table S8) of genes significantly upregulated in the inferior retina (Supplementary Table S6B) at each time point compared to DR controls. The top enriched terms are shown (FDR ≤ 0.05). [#]indicates terms not significantly enriched (FDR ≥ 0.05, $p \leq 0.05$). **(C)** Per spot cumulative gene expression levels (indicated by the heatmaps) of all significantly upregulates genes contained within indicated GO terms for spots overlapping the inferior retina. One representative replicate for DR controls and relevant PD sample where GO term is enriched are shown. DR – dim reared control, PD – photo-oxidative damage.

Supplementary Figure S10:

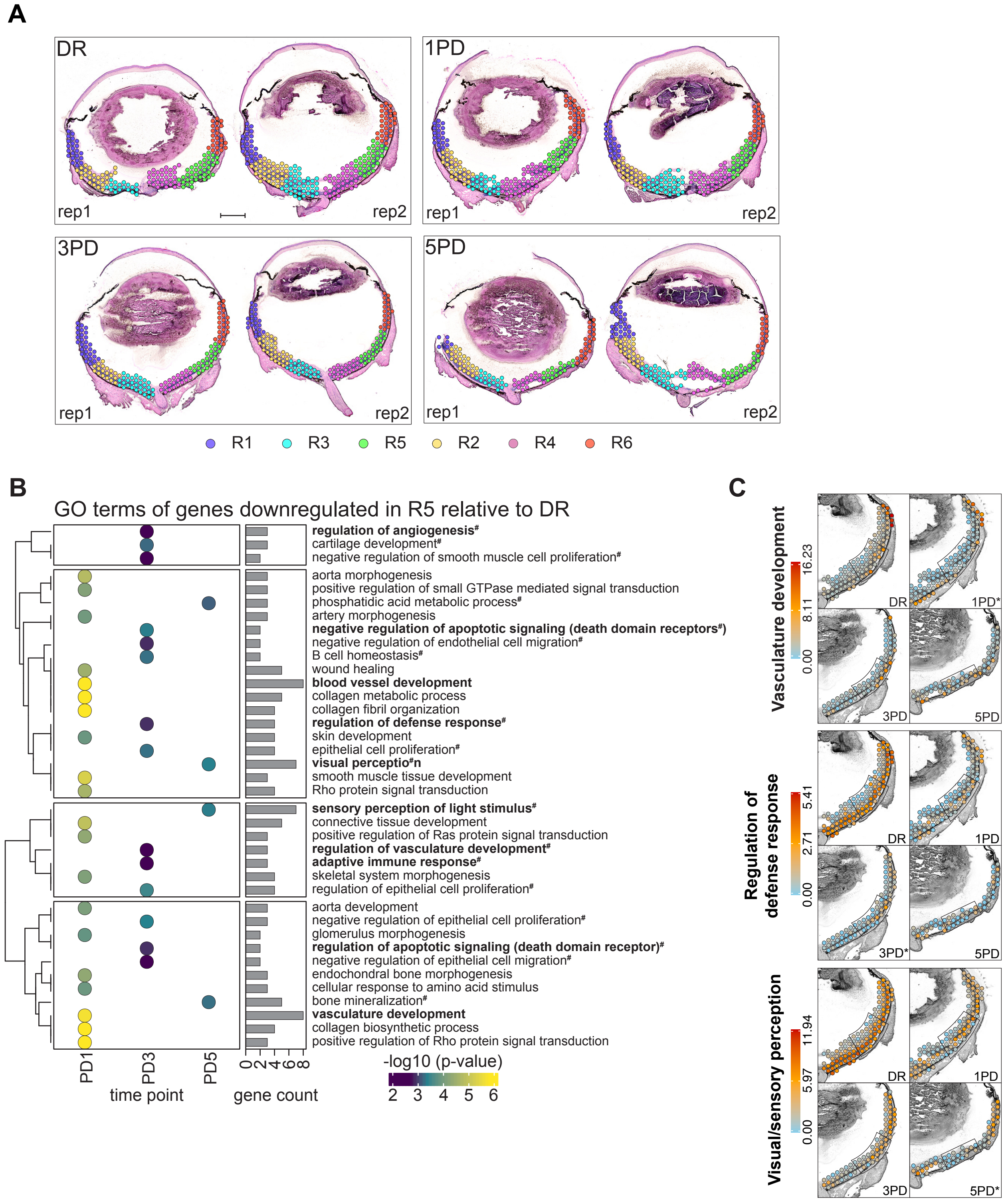

Supplementary Figure S10: (A) Segmentation of the retinal expression spots into six regions across all eight samples. **(B)** GO term enrichment (Supplementary Table S10D-F) analysis of genes significantly downregulated (Supplementary Table S10E) in R5 at each time point relative to DR controls. The top enriched terms are shown (FDR ≤ 0.05). #indicates terms not significantly enriched (FDR ≥ 0.05, *p* ≤ 0.05). **(C)** Per spot cumulative expression levels (indicated by the heatmaps) of all significantly downregulated genes contained within the indicated GO terms for spots overlapping the superior retina. Asterisk indicates the time point where downregulated genes were significantly associated with the GO term. One representative replicate for each time point is shown. Boxed regions indicate R5 of the superior retina. DR – dim reared control, PD – photo-oxidative damage.

Supplementary Figure S11:

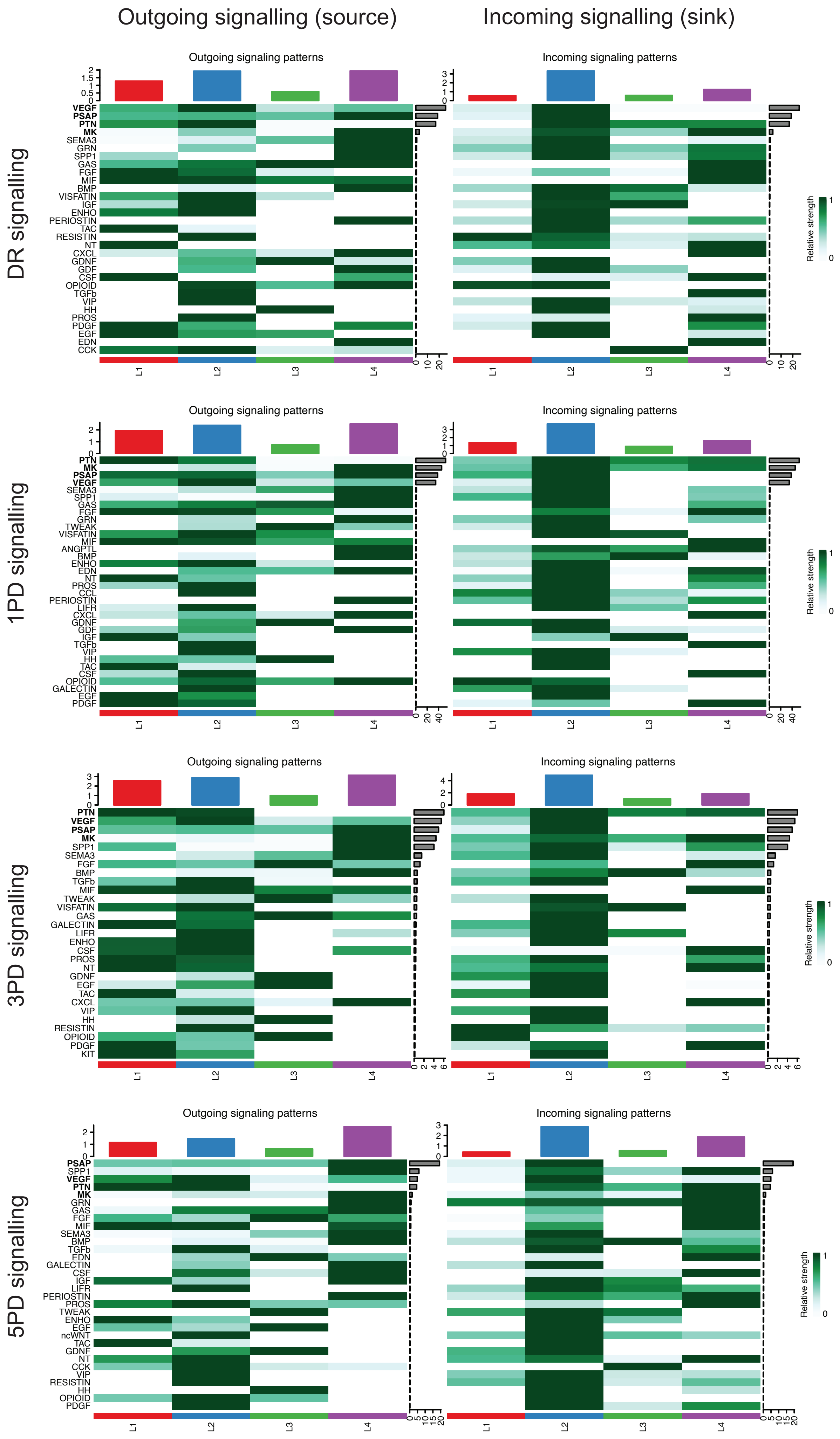

Supplementary Figure S11: CellChat analysis illustrating incoming (sink) and outgoing (source) signalling patterns for each superior retinal layer (L1-4) at each time point. Cumulative signalling strength (top bar chart) and number of interactions for each ligand (right bar chart) are shown. The heatmap indicates relative signalling strength for each ligand. DR – dim reared control, PD – photo-oxidative damage.

Supplementary Figure S12:

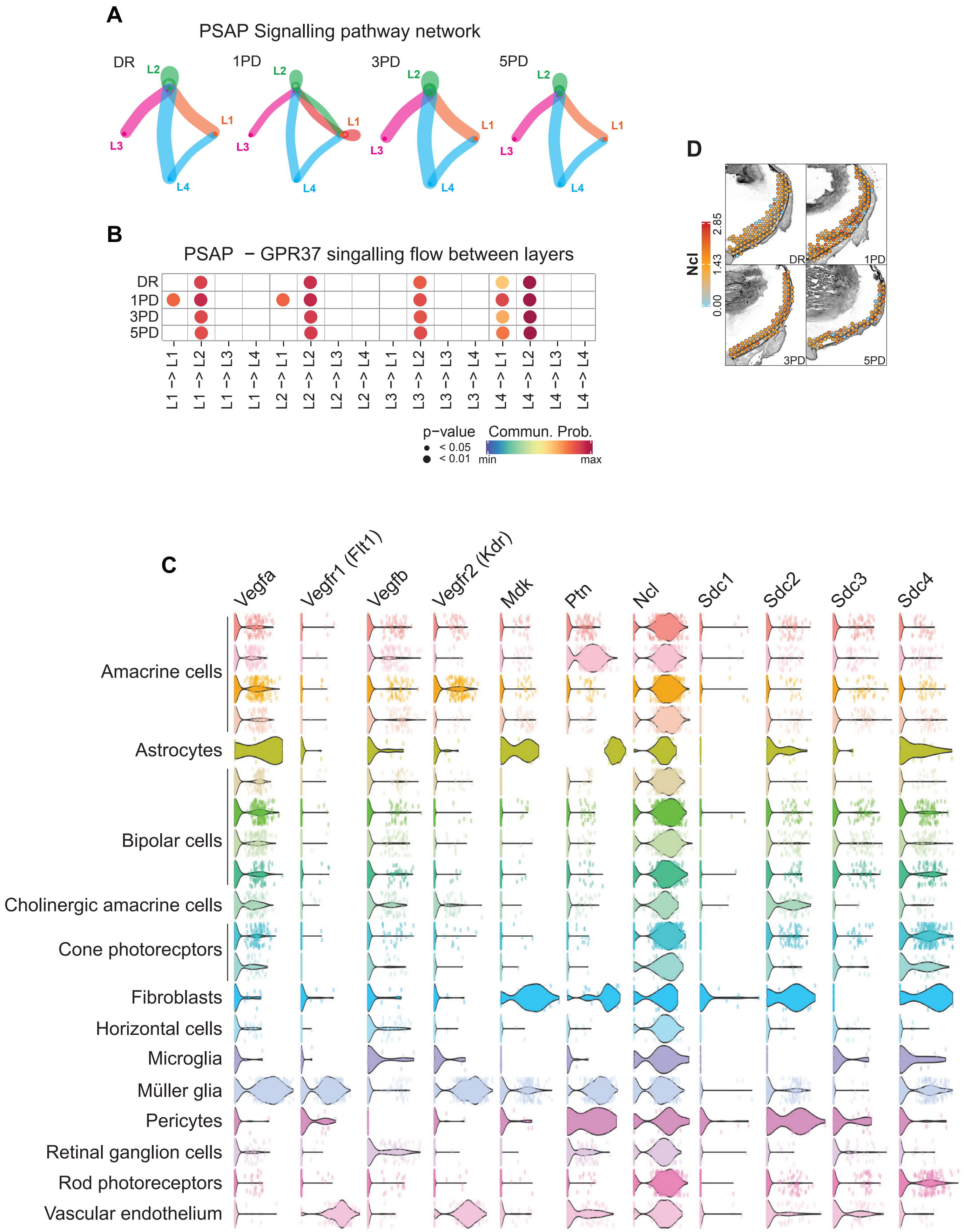

Supplementary Figure S12: (A) The inferred signalling network for the PSAP ligand across all layers at each time point. Line colours indicate layer of origin and line thickness indicates relative strength of communication. **(B)** CellChat analysis showing significant communication probabilities (heatmap) for the PSAP ligand with the GPR37 receptor. **(C)** Gene expression levels of key signalling pathway ligands and receptors within retinal cell types, determined using published retinal single cell sequencing data [7]. **(D)** Spatial mapping of per spot gene expression of the *Ncl* gene. One replicate per time point is shown. Heatmap indicates relative expression level. DR – dim reared control, PD – photo-oxidative damage

Supplementary References:

1 Rutar, M., Provis, J. M. & Valter, K. Brief exposure to damaging light causes focal recruitment of macrophages, and long-term destabilization of photoreceptors in the albino rat retina. *Curr Eye Res* **35**, 631-643 (2010). https://doi.org:10.3109/02713681003682925

2 Aggio-Bruce, R. *et al.* Inhibition of microRNA-155 Protects Retinal Function Through Attenuation of Inflammation in Retinal Degeneration. *Mol Neurobiol* **58**, 835-854 (2021). https://doi.org:10.1007/s12035-020-02158-z

3 Ni, Z. *et al.* SpotClean adjusts for spot swapping in spatial transcriptomics data. *bioRxiv*, 2021.2006.2011.448105 (2021). https://doi.org:10.1101/2021.06.11.448105

4 Bergenstrahle, J., Larsson, L. & Lundeberg, J. Seamless integration of image and molecular analysis for spatial transcriptomics workflows. *BMC Genomics* **21**, 482 (2020). https://doi.org:10.1186/s12864-020-06832-3

5 Pham, D. *et al.* stLearn: integrating spatial location, tissue morphology and gene expression to find cell types, cell-cell interactions and spatial trajectories within undissociated tissues. *bioRxiv*, 2020.2005.2031.125658 (2020). https://doi.org:10.1101/2020.05.31.125658

6 Hoang, T. *et al.* Gene regulatory networks controlling vertebrate retinal regeneration. *Science* **370** (2020). https://doi.org:10.1126/science.abb8598

7 Macosko, E. Z. *et al.* Highly Parallel Genome-wide Expression Profiling of Individual Cells Using Nanoliter Droplets. *Cell* **161**, 1202-1214 (2015). https://doi.org:10.1016/j.cell.2015.05.002
