# Supplementary material for: Spatial transcriptomics reveals regionally altered gene expression that drives retinal degeneration
Source: Commun Biol. 2025 Apr 18;8:629. doi: 10.1038/s42003-025-07887-2 (PMC12008306; doi:10.1038/s42003-025-07887-2)
Supplement: Supplementary file 2 — Description of Additional Supplementary Files [file 42003_2025_7887_MOESM2_ESM.pdf]

Description of Additional Supplementary Files

File name: Supplementary Data 1

Description: Supplementary Table S1 – S3

Table S1: Differentially expressed genes between biological replicates at each time point (FC ≥ 2; p-value ≤ 0.05)

Table S2: Retinal cell type-specific marker genes used for identifying majority cell type contribution to retinal spot clusters.

Table S3: Significant (FDR ≤ 0.05) GO terms of (A) genes upregulated in L1 compared to other layers; (B) genes downregulated in L1 compared to other layers; (C) genes upregulated in L2 compared to other layers; (D) genes downregulated in L2 compared to other layers; (E) genes upregulated in L3 compared to other layers; (F) genes downregulated in L3 compared to other layers; (G) genes upregulated in L4 compared to other layers; (H) genes downregulated in L4 compared to other layers.

File name: Supplementary Data 2

Description: Supplementary Table S4 – S5

Table S4: Differentially expressed genes in the superior retina compared to the inferior retina (p ≤ 0.05; FC ≥ 1.5) at each time point and across all eight samples (ALL). DR - dim-reared sample, PD - photo-oxidative damage paradigm, ALL - across all eight samples. NA - gene not differentially expressed in this comparison.

Table S5A: Significantly (FDR ≤ 0.05) enriched GO terms associated with genes significantly upregulated at (A) DR in the superior retina compared to the inferior retina; (B) 1PD in the superior retina compared to the inferior retina; (C) at 3PD in the superior retina compared to the inferior retina; (D) 5PD in the superior retina compared to the inferior retina. (E) Significantly (FDR ≤ 0.05) enriched GO terms associated with genes significantly downregulated at 5PD in the superior retina compared to the inferior retina. Enriched terms were reduced using Revigo and the most encompassing reduced term is indicated.

File name: Supplementary Data 3

Description: Supplementary Table S6 – S8

Table S6: (A) Differentially expressed genes in the superior retina at 1PD, 3PD and 5PD compared to DR controls (p ≤ 0.05; FC ≥ 1.5). (B) Differentially expressed genes in the inferior retina at 1PD, 3PD and 5PD compared to DR controls (p ≤ 0.05; FC ≥ 1.5). DR - dim-reared sample, PD - photo-oxidative damage paradigm. NA - gene not differentially expressed in this comparison.

Table S7: (A) Significantly (FDR ≤ 0.05) enriched GO terms associated with genes significantly upregulated in the superior at 1PD compared to DR controls. Enriched terms

were reduced using Revigo and the most encompassing reduced term is indicated. (B) Top 20 GO terms associated with genes significantly upregulated in the superior at 3PD compared to DR controls. Note: only the top three terms are significantly enriched. Revigo reduction was not performed. (C) Significantly (FDR ≤ 0.05) enriched GO terms associated with genes significantly upregulated in the superior at 5PD compared to DR controls. Enriched terms were reduced using Revigo and the most encompassing reduced term is indicated.

Table S8: (A) Top 20 enriched GO terms associated with genes significantly upregulated in the inferior at 1PD compared to DR controls. Note: only the top seven terms are significantly enriched. Enriched terms were reduced using Revigo and the most encompassing reduced term is indicated. (B) Significantly (FDR ≤ 0.05) enriched GO terms associated with genes significantly upregulated in the inferior at 3PD compared to DR controls. Enriched terms were reduced using Revigo and the most encompassing reduced term is indicated. (C) Significantly (FDR ≤ 0.05) enriched GO terms associated with genes significantly upregulated in the inferior at 5PD compared to DR controls. Enriched terms were reduced using Revigo and the most encompassing reduced term is indicated.

File name: Supplementary Data 4

Description: Supplementary Table S9 – S11

Table S9: Genes differentially expressed genes at 1PD, 3PD, 5PD compared to DR controls (p ≤ 0.05; FC ≥ 1.5) in (A) retinal region 1 (R1); (B) retinal region 2 (R2); (C) retinal region 3 (R3); (D) in retinal region 4 (R4); (E) retinal region 5 (R5); (F) retinal region 6 (R6). DR - dim-reared sample, PD - photo-oxidative damage paradigm. NA - gene not differentially expressed in this comparison.

Table S10: Significantly (FDR ≤ 0.05) enriched GO terms associated with (A) genes significantly upregulated in region 5 (R5) at 1PD compared to DR controls; (B) genes significantly upregulated in region 5 (R5) at 3PD compared to DR controls; (C) genes significantly upregulated in region 5 (R5) at 5PD compared to DR controls; Enriched terms were reduced using Revigo and the most encompassing reduced term is indicated. Significantly (FDR ≤ 0.05) enriched GO terms associated with (D) genes significantly downregulated in region 5 (R5) at 1PD compared to DR controls; (E) genes significantly downregulated in region 5 (R5) at 3PD compared to DR controls; (F) significantly downregulated in region 5 (R5) at 5PD compared to DR controls. Enriched terms were reduced using Revigo and the most encompassing reduced erm is indicated. Note: in E and F none of the terms are significantly enriched (FDR ≤ 0.05); the top 20 GO terms associated with significantly dysregulated genes are shown. Revigo reduction was not performed.

Table S11: Significantly (FDR ≤ 0.05) enriched GO terms associated with (A) genes significantly upregulated in region 4 (R4) at 1PD compared to DR controls; (B) genes significantly upregulated in region 4 (R4) at 3PD compared to DR controls; (C) genes significantly upregulated in region 4 (R4) at 5PD compared to DR controls; (D) genes significantly downregulated in region 4 (R4) at 1PD compared to DR controls; (E) genes significantly downregulated in region 4 (R4) at 3PD compared to DR controls. Enriched terms were reduced using Revigo and the most encompassing reduced term is indicated. Note: in A, D and E none of the terms are significantly enriched (FDR ≤ 0.05); the top 20 GO

terms are shown. Revigo reduction was not performed. No GO terms were significantly ($p \leq 0.05$) enriched in genes significantly downregulated in region 4 (R4) at 5PD compared to DR controls.

File name: Supplementary Data 5

Description: Supplementary Table S12 – S14

Table S12: Differentially expressed genes ($p \leq 0.05$; $FC \geq 1.5$) in (A) retinal ganglion cell layer (GCL; layer 1 (L1)) at 1PD, 3PD and 5PD relative to DR controls; (B) retinal interneuron layer (layer 2 (L2)) at 1PD, 3PD and 5PD relative to DR controls; (C) Table S12C; (D) retinal pigment epithelial/choroid layer (RPE/choroid; layer 4 (L4)) at 1PD, 3PD and 5PD relative to DR controls. DR - dim reared sample, PD - photo-oxidative damage paradigm. NA - gene not differentially expressed in this comparison.

Table S13: Significantly ($FDR \leq 0.05$) enriched GO terms associated with (A) genes significantly downregulated in layer 1 (GCL) at 1PD compared to DR controls; (B) genes significantly downregulated in layer 1 (GCL) at 3PD compared to DR controls; (C) genes significantly downregulated in layer 1 (GCL) at 5PD compared to DR controls; (D) genes significantly downregulated in layer 2 (INL) at 1PD compared to DR controls; (E) genes significantly downregulated in layer 2 (INL) at 3PD compared to DR controls; (F) genes significantly downregulated in layer 2 (INL) at 5PD compared to DR controls; (G) genes significantly downregulated in layer 3 (Photoreceptors) at 1PD compared to DR controls; (H) genes significantly downregulated in layer 3 (Photoreceptors) at 3PD compared to DR controls; (I) genes significantly downregulated in layer 3 (Photoreceptors) at 5PD compared to DR controls; (J) genes significantly downregulated in layer 4 (RPE/choroid) at 1PD compared to DR controls; (K) genes significantly downregulated in layer 4 (RPE/choroid) at 3PD compared to DR controls; (L) genes significantly downregulated in layer 4 (RPE/choroid) at 5PD compared to DR controls. Enriched terms were reduced using Revigo and the most encompassing reduced term is indicated. Note: in A and E no GO terms were significantly enriched.

Table S14: Significantly ($FDR \leq 0.05$) enriched GO terms associated with (A) genes significantly upregulated in layer 1 (GCL) at 1PD compared to DR controls; (B) genes significantly upregulated in layer 1 (GCL) at 3PD compared to DR controls; (C) genes significantly upregulated in layer 1 (GCL) at 5PD compared to DR controls; (D) genes significantly upregulated in layer 2 (INL) at 1PD compared to DR controls; (E) genes significantly upregulated in layer 2 (INL) at 3PD compared to DR controls; (F) genes significantly upregulated in layer 2 (INL) at 5PD compared to DR controls; (G) genes significantly upregulated in layer 3 (Photoreceptor) at 1PD compared to DR controls; (H) genes significantly upregulated in layer 3 (Photoreceptor) at 3PD compared to DR controls; (I) genes significantly upregulated in layer 3 (Photoreceptor) at 5PD compared to DR controls; (J) genes significantly upregulated in layer 4 (RPE/choroid) at 1PD compared to DR controls; (K) genes significantly upregulated in layer 4 (RPE/choroid) at 3PD compared to DR controls; (L) genes significantly upregulated in layer 4 (RPE/choroid) at 5PD compared to DR controls. Enriched terms were reduced using Revigo and the most encompassing reduced term is indicated.
