# Supplementary material for: Spatial transcriptomics reveals regionally altered gene expression that drives retinal degeneration
Source: Commun Biol. 2025 Apr 18;8:629. doi: 10.1038/s42003-025-07887-2 (PMC12008306; doi:10.1038/s42003-025-07887-2)
Supplement: Supplementary file 8 — Reporting Summary [file 42003_2025_7887_MOESM8_ESM.pdf]

nature portfolio

Corresponding author(s): Riccardo Natoli
Ulrike Schumann

Last updated by author(s): 03.02.2025

Reporting Summary

Nature Portfolio wishes to improve the reproducibility of the work that we publish. This form provides structure for consistency and transparency in reporting. For further information on Nature Portfolio policies, see our Editorial Policies and the Editorial Policy Checklist.

Statistics

For all statistical analyses, confirm that the following items are present in the figure legend, table legend, main text, or Methods section.

n/a	Confirmed	
☐	☒	The exact sample size (*n*) for each experimental group/condition, given as a discrete number and unit of measurement
☐	☒	A statement on whether measurements were taken from distinct samples or whether the same sample was measured repeatedly
☐	☒	The statistical test(s) used AND whether they are one- or two-sided<br>*Only common tests should be described solely by name; describe more complex techniques in the Methods section.*
☒	☐	A description of all covariates tested
☐	☒	A description of any assumptions or corrections, such as tests of normality and adjustment for multiple comparisons
☐	☒	A full description of the statistical parameters including central tendency (e.g. means) or other basic estimates (e.g. regression coefficient) AND variation (e.g. standard deviation) or associated estimates of uncertainty (e.g. confidence intervals)
☒	☐	For null hypothesis testing, the test statistic (e.g. *F*, *t*, *r*) with confidence intervals, effect sizes, degrees of freedom and *P* value noted<br>*Give P values as exact values whenever suitable.*
☒	☐	For Bayesian analysis, information on the choice of priors and Markov chain Monte Carlo settings
☐	☒	For hierarchical and complex designs, identification of the appropriate level for tests and full reporting of outcomes
☒	☐	Estimates of effect sizes (e.g. Cohen's *d*, Pearson's *r*), indicating how they were calculated

Our web collection on statistics for biologists contains articles on many of the points above.

Software and code

Policy information about availability of computer code

Data collection	The open source software Zen was used as part of data collection.
Data analysis	All source code developed for this work is accessible on Github https://github.com/jiayuwen/Spatial_Retina<br>Open source software used for data analysis in this project include SpaceRanger, SpotClean, STUtility, stLearn, Harmony, Seurat v3, Spotlight, edgeR, clusterProfiler, REVIGO, CellChat and Zen

For manuscripts utilizing custom algorithms or software that are central to the research but not yet described in published literature, software must be made available to editors and reviewers. We strongly encourage code deposition in a community repository (e.g. GitHub). See the Nature Portfolio guidelines for submitting code & software for further information.

Data

Policy information about availability of data

All manuscripts must include a data availability statement. This statement should provide the following information, where applicable:

- Accession codes, unique identifiers, or web links for publicly available datasets
- A description of any restrictions on data availability
- For clinical datasets or third party data, please ensure that the statement adheres to our policy

The dataset created in this study has been submitted to NCBI Gene Expression Omnibus (GEO) under accession number GSE248517 with a reviewer access code provided in the letter to the editor.

Research involving human participants, their data, or biological material

Policy information about studies with human participants or human data. See also policy information about sex, gender (identity/presentation), and sexual orientation and race, ethnicity and racism.

Reporting on sex and gender	NA
Reporting on race, ethnicity, or other socially relevant groupings	NA
Population characteristics	NA
Recruitment	NA
Ethics oversight	NA

Note that full information on the approval of the study protocol must also be provided in the manuscript.

Field-specific reporting

Please select the one below that is the best fit for your research. If you are not sure, read the appropriate sections before making your selection.

☒ Life sciences ☐ Behavioural & social sciences ☐ Ecological, evolutionary & environmental sciences

For a reference copy of the document with all sections, see nature.com/documents/nr-reporting-summary-flat.pdf

Life sciences study design

All studies must disclose on these points even when the disclosure is negative.

Sample size	The study utilises biological replicates for each time point (n=2). No sample size calculations were performed. This study involves highly cost intensive spatial transcriptomics technology and as such biological replication was deemed sufficient.
Data exclusions	Expression spots with low expression level (<500 features and >30% mitochondrial contribution) were excluded.
Replication	We reproducibility between replicates by assessing differential expression between replicates samples, which was found to be negligible.
Randomization	Allocation of mice into the study was random.
Blinding	Researchers were not blinded to the samples. this is a differential expression analysis between time points, blinding would not have allowed appropriate data analysis.

Reporting for specific materials, systems and methods

We require information from authors about some types of materials, experimental systems and methods used in many studies. Here, indicate whether each material, system or method listed is relevant to your study. If you are not sure if a list item applies to your research, read the appropriate section before selecting a response.

Materials & experimental systems

n/a	Involved in the study	
☐	☒	Antibodies
☒	☐	Eukaryotic cell lines
☒	☐	Palaeontology and archaeology
☐	☒	Animals and other organisms
☒	☐	Clinical data
☒	☐	Dual use research of concern
☒	☐	Plants

Methods

n/a	Involved in the study	
☒	☐	ChIP-seq
☒	☐	Flow cytometry
☒	☐	MRI-based neuroimaging

Antibodies

Antibodies used	IBA antibody: Sapphire Bioscience, Cat# 111-IBA1-0022, Lot:AIF87967980<br>GFAP antibody: Invitrogen, Cat# MA5-12023, Lot is YK4112203

Validation	The primary antibodies are validated for Immunohistochemistry experiments for mouse tissue by the manufacturer.

Animals and other research organisms

Policy information about studies involving animals; ARRIVE guidelines recommended for reporting animal research, and Sex and Gender in Research

Laboratory animals	Wild-type C57BL6/J mice of 6 week of age were used for this study
Wild animals	NA
Reporting on sex	Male mice were used in this study and findings only apply to the male sex. We did not include both sexes in the study as only biological replicates were performed and this might have led to lower data reproducibility negating the opportunities that arise from duplication.
Field-collected samples	NA
Ethics oversight	ANU Animal Experimentation Ethics Committee (Ethics ID: A2020/41) approved the study

Note that full information on the approval of the study protocol must also be provided in the manuscript.

Plants

Seed stocks	NA
Novel plant genotypes	NA
Authentication	NA
